# Determination of the Role of DDX3 a Factor Involved in Mammalian RNAi Pathway Using an shRNA-Expression Library

**DOI:** 10.1371/journal.pone.0059445

**Published:** 2013-03-19

**Authors:** Vivi Kasim, Shourong Wu, Kazunari Taira, Makoto Miyagishi

**Affiliations:** 1 Key Laboratory of Biorheological Science and Technology, Ministry of Education, College of Bioengineering, Chongqing University, Chongqing, China; 2 Department of Chemistry and Biotechnology, School of Engineering, The University of Tokyo, Tokyo, Japan; 3 Tokyo University and Graduate School of Social Welfare, Tokyo, Japan; 4 Molecular Composite Medicine Research Group, Biomedical Research Institute, National Institute of Advanced Industrial Science and Technology (AIST), Tsukuba, Japan; French National Center for Scientific Research - Institut de biologie moléculaire et cellulaire, France

## Abstract

RNA interference (RNAi) is an endogenous RNA-destruction phenomenon induced by certain double-stranded RNAs (dsRNAs). In RNAi, dsRNAs are processed into small interfering RNAs (siRNAs) which in turn trigger the cleavage of the target mRNA. Here, using a short hairpin RNA-expression library, we identified a DEAD-box helicase 3, DDX3, as an essential factor involved in RNAi pathway and revealed that DDX3 is colocalized with Ago2, an essential factor in RNAi pathway that cleaves target mRNA. Results of experiments with a dominant negative mutant of DDX3 further confirmed that this factor affects the RNAi activity. Together, DDX3 functions to assure mammalian RNAi pathway. Together, our results indicate that DDX3 is a new key molecule to understand the molecular mechanism underlying RNAi pathway in mammals.

## Introduction

RNA interference (RNAi) is an endogenous RNA-destruction phenomenon induced by certain double-stranded RNAs (dsRNAs) found in various organisms including plants, *C. elegans*, *Drosophila* and mammals [1]–[4]. In RNAi, dsRNAs are processed to yield 21–23 bps small interfering RNAs (siRNAs) in reactions catalyzed by Dicer [5]–[8]. The siRNAs are then processed into single-stranded RNAs, which subsequently trigger the cleavage of the target mRNA in a reaction that is mediated by RNA-Induced Silencing Complex (RISC) [9], [10].

Gene silencing induced by RNAi pathway involved several factors. In mammals, firstly, Dicer, a type III RNase having RNA binding domain, endonuclease domain and helicase domain, was identified as one of the key factors in RNAi. Dicer triggers the processing of dsRNAs into siRNAs [5]. Later on, Argonaute 2 (Ago2) was identified as a component of mammalian RISC which cleaves the target mRNA [11]. However, other critical factors in RNAi remain to be unequivocally identified. Identification of factors involved in the RNAi pathway is one of the essential parts of elucidation of the whole gene silencing mechanisms.

In order to elucidate other factors involved in the RNAi pathway, we generated short hairpin RNA (shRNA)-expression libraries against genes which have helicase domains, RNA binding domains and/or nuclease domains. Here, as the first effort, we used the shRNA-expression library to screen genes with helicase domains, and identified DDX3 as one of the factors that play a critical role in RNAi pathway.

## Materials and Methods

### Culture and Transfection of Cells and Assays of the Expression of Reporter Genes

HeLa S3 cells were cultured in Dulbecco’s modified Eagle’s medium (DMEM) supplemented with 1% antibiotics and 10% fetal bovine serum. For the screening of our library, we plated HeLa S3 cells in 96-well plates and transfected them with 300 ng of individual shRNA vectors having puromycin resistant gene by using the Effectene™ reagent (Qiagen, Valencia, CA). Then 24 h after transfection, to eliminate untransfected cells, we incubated the cells in the presence of 1 µg/ml puromycin for 48 h, and replated them into a new plate. We either further cultured the cells for the time indicated, or directly performed second transfections using Lipofectamine™ 2000 (Invitrogen, Carlsbad, CA) mixed with 2.5 ng of shRNA vector against firefly luciferase, 25 ng of firefly luciferase-expression vector (pGL3-control vector; Promega, Madison, WI) and 2.5 ng of *Renilla*-luciferase expression plasmid (pRL-CMV; Promega). Then 8 to 24 h after the second transfection, we measured the activities of firefly and *Renilla* luciferase with the Dual Luciferase Assay System (Promega). For the assay using EGFP reporter gene, we used an shRNA vector directed against EGFP, an shRNA vector directed against DsRed and an empty shRNA vector, with TTTTTTT inserted immediately downstream of the site initiation of transcription, served as negative controls. To ensure transfection of equal amounts of DNA, empty plasmids were added at appropriate levels in each transfection. For fluorescence analysis, HeLa S3 cells were transfected with 1 µg of shRNA vector directed against DDX3 or the empty shRNA vector and selected with puromycin as described above. Then the cells were cotransfected with an shRNA vector against EGFP (30 ng) or DsRed2 (100 ng), 200 ng of pEGFP (BD Biosciences, Palo Alto, CA) and 50 ng of pDsRed2 (BD Biosciences). Forty-eight hours after transfection, cells were examined with a confocal microscope (Leica, Wetzlar, Germany).

### Construction of shRNA Vectors

The shRNA library was generated as described previously [12]. The DDX3-expression vector was generated by RT-PCR-based subcloning into pcDNA3. Mutation in DDX3 was generated by PCR-based site-directed mutagenesis. The sequences of the insert in shRNA vectors against firefly luciferase, DsRed2, and EGFP were 5′-GTG CGT TGT TGG TGT TAA TCC TTC AAG AGA GGG TTG GCA CCA GCA GCG CAC TT -3′, 5′-GTG GGG GCG TGT GGT GAA CTT GTG TGC TGT CCA AGT TCA TCA CGC GCT CCC ACT T -3′, and 5′-GGC TAT GTC TAG GAG TGT ACC GTG TGC TGT CGG TGC GCT CCT GGA CGT AGC CTT-3′, respectively. The sequences of the insert in shRNA vectors against DDX3 and Ago2 were as follows: DDX3 site 1, 5′-GAT TTA AGG GGG AGG TCT AAC GTG TGC TGT CCG TTA GAC TTC CCT CTT GAA TCT TT-3′; DDX3 site 21, 5′-GCT TCA GAT TTG TAG GAT AAC GTG TGC TGT CCG TTA TTC TAC GAA TCT GAG GCT TT-3′; DDX3 site 32, 5′-GTG ATA AAT TAG ATG GAG AAC GTG TGC TGT CCG TTC TTC ATC TGA TTT GTC ACT TT-3′; DDX3 site 4 3, 5′-GAG GGA ATT GGG ATA GCA TAC GTG TGC TGT CCG TAT GCT GTC CCA GTT CTC TCT TT-3′; DDX3 site 54, 5′-GCT GCA AAC GAT ACC TAA TAC GTG TGC TGT CCG TAT TGG GTA TTG TTT GCG GCT TT-3′; and Ago2, 5′-GCT TGA AGG TCA ATG TCA AAC GTG TGC TGT CCG TTT GAC GTT GAT CTT CAG GCT TT-3′. Details of all methods are available on request.

### Immunohistochemical Analysis

Immunohistochemical analysis was performed as described previously [13]. Mouse monoclonal anti-DCP1a (clone 3G4) was purchased from Wako Pure Chemical Industries (Osaka, Japan). Mouse anti-HA antibody (12CA5) was purchased from Roche Diagnostics (Indianapolis, IN,. U.S.A). Rabbit anti-Flag M2 antibody, Anti-mouse IgG (H+L), F(ab′)2 Fragment (Alexa Fluor 488) and Anti-rabbit IgG (H+L), F(ab′)2 Fragment (Alexa Fluor 555) were purchased from Cell Signaling Technology, Inc. (Boston, MA, USA).

### Statistical Analysis

Results are presented as Mean and S.D. Statistical comparisons of values were made using two-tailed Student’s *t* tests. Statistical significance was defined as *P*<0.05 (*, *P*<0.05).

## Results and Discussion

**Figure 1:**
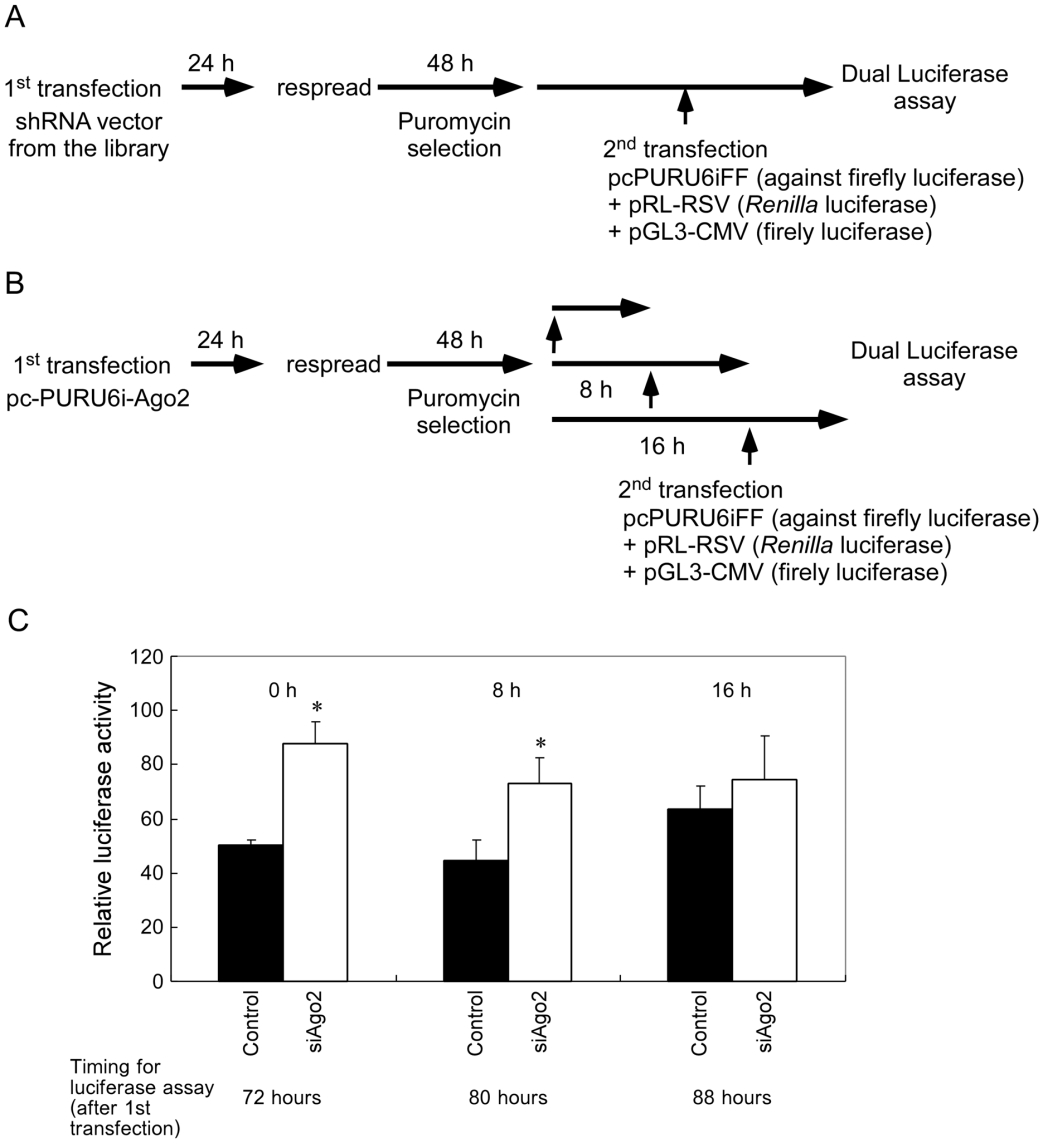
Confirmation of the screening system of the shRNA library by using the shRNA directed against human Ago2 (pcPURU6i-Ago2). (A) General schematic diagram of the screening procedure. (B) Schematic diagram of the optimization for the screening condition using Ago2 as a positive control. (C) The inhibitory effects of the shRNA directed against human Ago2 (pcPURU6i-Ago2) on RNAi. The second transfection was performed at 72 h, 80 h and 88 h after the first transfection, and the luciferase activity was determined at 80 h, 88 h and 96 h after the first transfection. Data was shown as mean and S.D. (n = 3). *P<0.05.

In an effort to identify factors that are involved in RNAi, we generated shRNA-expression vector libraries directed against all known human genes with helicase domains, RNA binding domains and/or nuclease domains [12]. Then we screened genes that code helicase domains to identify RNAi-disrupting clones using a dual-luciferase reporter system. The general schematic diagram of the screening procedure is shown in Figure 1A. In our system, we cotransfected an shRNA vector directed against firefly luciferase and two vectors that are firefly luciferase reporter and *Renilla* luciferase reporter into cells previously transfected with shRNA-expression vector from our library. We reasoned that, if essential genes for RNAi were disrupted by a specific shRNA vector(s) against RNAi-related genes, we would detect the restoration of the activity of the firefly luciferase. The *Renilla* luciferase reporter vector was used to normalize the luciferase activities.

In our screening system, we used shRNA vector against human Ago2 as a positive control. As Ago2 is an essential factor in RNAi pathway, utilization of a time lag between mRNA suppression and RNAi effect is considered to be needed. Therefore, firstly, to evaluate an RNAi-disrupting effect using Ago2 and to optimize the screening conditions for Ago2, we set up a time course experiment as shown in Figure 1B. In other words, it is crucial to determine an appropriate time point for the second transfection, *i.e.*, the transfection of shRNA vector directed against firefly luciferase, firefly luciferase reporter and *Renilla* luciferase reporter, so that the effect of the shRNA against Ago2 could be observed. As shown in Figure 1C, transfection of the shRNA vector against Ago2 in HeLa S3 cells resulted in significant disruption of RNAi effect when the second transfection was performed immediately or with additional 8 h culture time after the 48 h puromycin selection, *i.e.*, 72 or 80 h after the first transfection. However, a lesser effect could be observed when the additional culture time was prolonged to 16 h, *i.e.*, 88 h after the first transfection. It should be noted that since the condition for screening was set using Ago2 as a positive control, other critical factors in RNAi pathway might require different optimal conditions depending on their protein stabilities.

Based on this result and subsequent preliminary experiments, we determined the optimal experimental condition as follows. HeLa S3 cells were transfected with individual shRNA-expression vector from our library in 96-well plates. Then, 24 h after transfection, we performed puromycin selection for 48 h to eliminate untransfected cells. We plated surviving cells in wells of a new 96-well plate with equal numbers in each well and transfected the cells with an shRNA vector against firefly luciferase, a firefly luciferase-expression vector, and a *Renilla* luciferase-expression vector. We examined the RNAi activity of the shRNA vector directed against the firefly luciferase transcript by measuring luciferase activities after the second transfection. We postulated that, under these conditions, participants in RNAi would be suppressed in cells transfected with shRNA vectors against genes involve in RNAi pathway.

**Figure 2:**
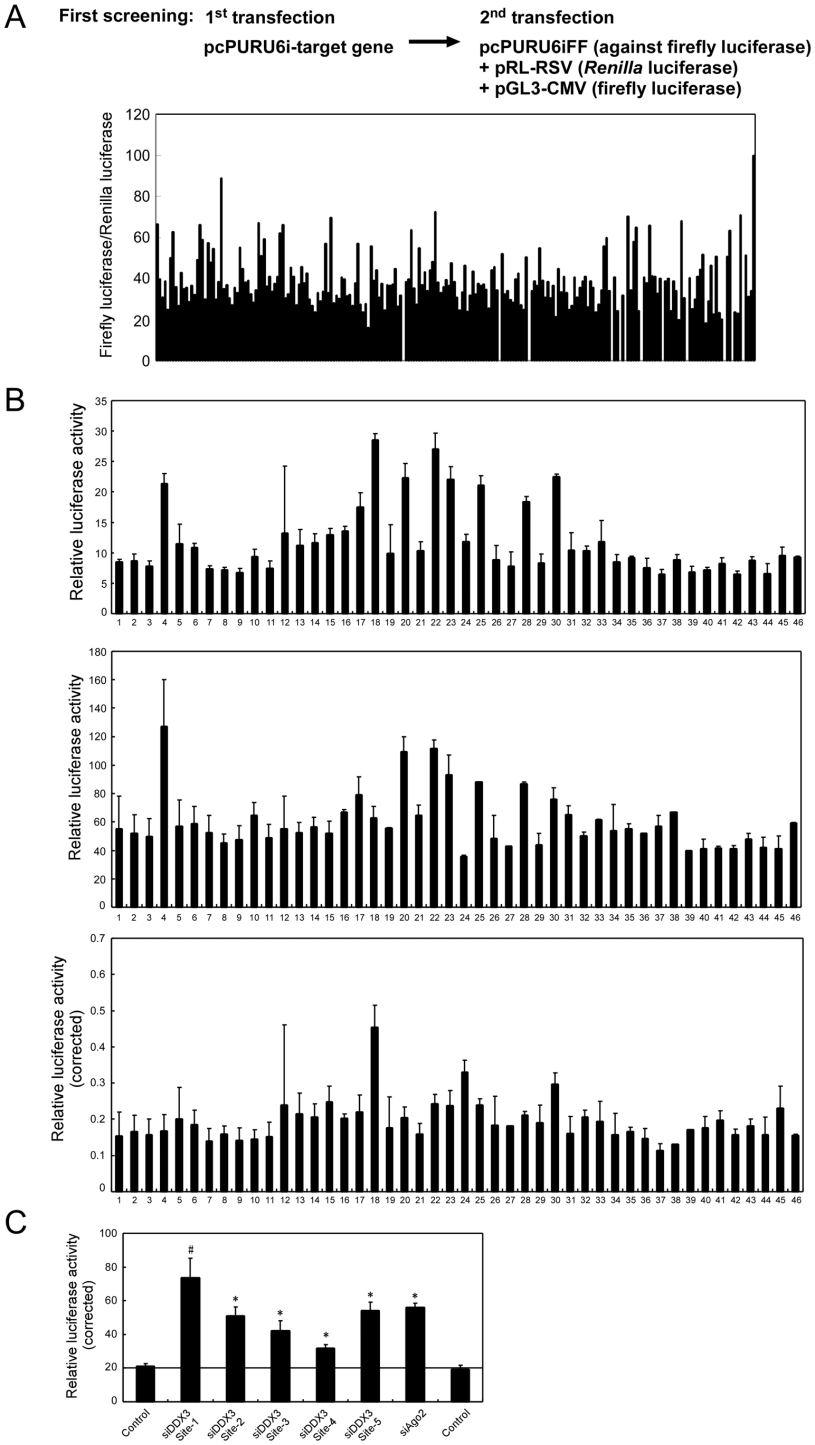
Screening of the shRNA library for identifying essential factors involved in RNAi pathway. (A) The first screening scheme and results of the shRNA library directed against human helicases and RNAi-related genes. The screening was performed in duplicate. (B) Results of control experiments for inhibitory effects on RNAi. Luciferase data before correction (upper panel), results of control experiments (middle panel), and data after correction (bottom panel) are shown. Clones #1, #2 and #46 are empty shRNA vector (negative control), and clone #24 is Ago2 (positive control). (C) Multi-target analysis using five shRNA vectors directed against various sites in DDX3 mRNA. #, clone that showed reduced cell proliferation. Data was shown as mean and S.D. (n = 3). *P<0.05.

In the first screening, we picked up 43 positive clones out of a total of 432 clones (216 genes), including clones that exhibited weak RNAi-inhibitory activity (Figure 2A). Despite the fact that we had created our shRNA library with emphasis on (i) high-level knockdown activity; (ii) the intracellular stability of the vector; (iii) avoidance of the interferon responses; and (iv) inactivation of the sense strand to minimize off-target effects [12], [14], detailed analysis of the 43 positive clones revealed a number of false positives. In some cases, the first transfection of cells with individual shRNA clones from our library affected the relative ratio of firefly/*Renilla* luciferase activity for unknown reasons. To compensate for this effect, we performed another set of transfection experiments in which we used an empty shRNA vector instead of the shRNA vector directed against firefly luciferase in the second transfection (Figure 2B, second row). Thus control experiments (Figure 2B, second row) allowed normalization of the varying luciferase activities that arose from the first transfection of cells with clones in the shRNA library (Figure 2B, top) to yield corrected relative luciferase activities (Figure 2B, bottom).

The second screening for positive clones, using the above-described normalization, revealed that at least two shRNA clones had strong RNAi-inhibitory activity (Figure 2B, bottom) compare to empty shRNA vectors (#1, #2 and #46). The most effective clone was the shRNA vector directed against a DEAD-box helicase, DDX3 (Figure 2B, bottom, #18), and the second effective clone was the shRNA vector against the human Ago2 (Figure 2B, bottom, #24). In subsequent analysis, we focused on the DDX3 for further analysis.

Although it is generally believed that suppression of gene expression by siRNA is specific, siRNA can cause nonspecific effects, such as off-target effect and interferon response that cause down regulation of protein expressions in general at the transcriptional and translational levels. To determine whether the positive clone for DDX3 might act nonspecifically, we designed four additional shRNA vectors directed against various sites in the mRNA for DDX3 and examined their RNAi-inhibitory effects. As shown in Figure 2C, three out of five shRNA vectors directed against DDX3 had a significant RNAi-inhibitory effect (the shRNA vector against DDX3 at site 1 showed reduced cell proliferation). This multi-target experiment suggested that knockdown of DDX3 by the shRNA vectors blocked RNAi pathway via specific rather than off-target effects. Furthermore, the facts that the shRNAs against DDX3 did not suppress the activity of Renilla luciferase used as an internal control (data not shown), and that the other shRNA vectors did not show RNAi-inhibitory effect (Figure 2B) suggested that the shRNA vectors did not trigger the interferon response. Together, these results indicated that the RNAi-inhibitory effect of DDX3 is specific.

**Figure 3:**
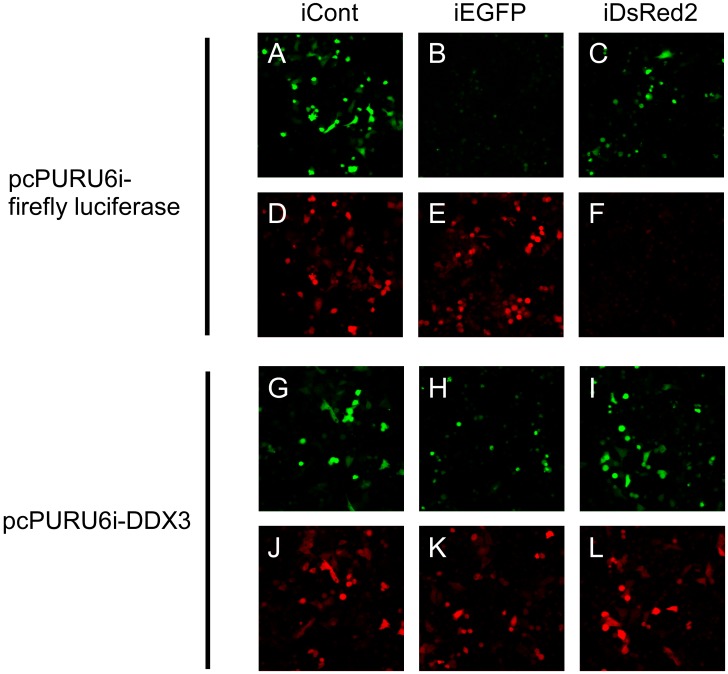
Suppression of the expression of DDX3 reduces the effects of RNAi on expression of genes for GFP and DsRed. HeLa S3 cells were transfected with an shRNA vector against firefly luciferase (A–F) or an shRNA vector against DDX3 (G–L) and selected by exposure to puromycin. Then, the cells were cotransfected with an shRNA vector directed against GFP or DsRed, a GFP-expression vector and a DsRed-expression vector. Finally, 48 h after the second transfection, the expression of GFP and DsRed was monitored by fluorescence microscopy.

We further confirmed the specificity in similar experiments with other reporter systems, as shown in Figure 3. In control cells, the shRNA vector against DsRed2 and the shRNA vector directed against EGFP (enhanced green fluorescence protein) suppressed the expression of their respective target genes (Figure 3, A–F). By contrast, in DDX3-knockdown cells, these shRNA vectors failed to suppress the expression of their target genes (Figure 3, G–L). Thus, this set of experiments confirmed our hypothesis that DDX3 is indispensable for RNAi.

**Figure 4:**
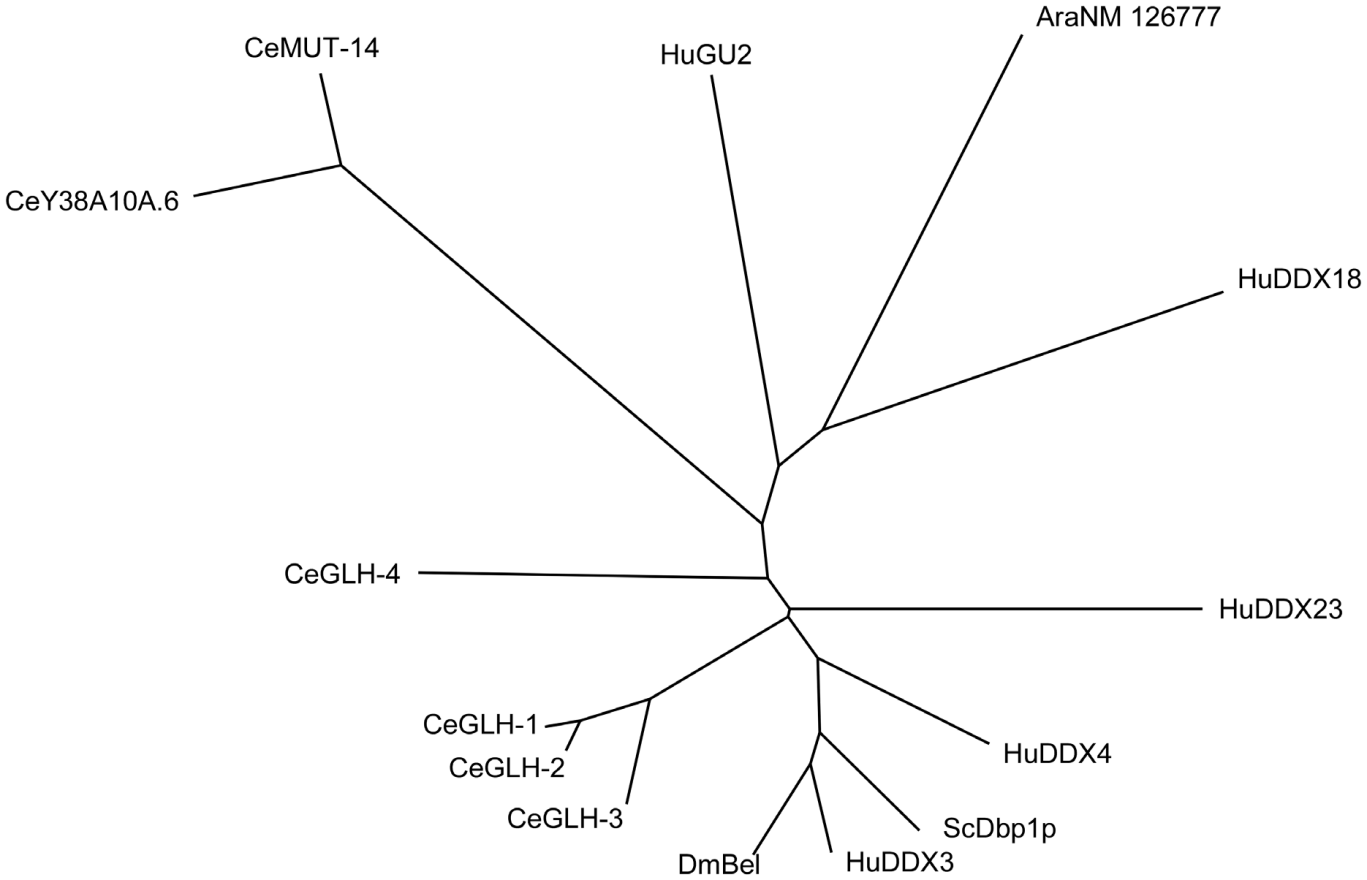
Phylogenetic tree showing homologs of DDX3 in selected species, formatted with TreeView (Page, 1996) using the ClustalW output. Accession numbers of sequences used in the calculation includes: HuDDX3, human DDX3; NP_076829, HuDDX4; human DDX4, NP_077726; HuDDX18, human DDX18, NP_006764; HuDDX23, human DDX23, NP_004809; HuGU2, human GU2, NP_076950; DmBel, *D. melanogaster* Bel, NP_536783; CeMut-14, *C. elegans* MUT-14, NP_504490; CeGLH-1, *C. elegans* GLH-1, NP_491963; CeGLH-2, *C. elegans* GLH-2, NP_491876; CeGLH-3, *C. elegans* GLH-3, NP_491681; CeGLH-4, *C. elegans* GLH-4, NP_491207; CeY38A10A.6, *C. elegans* CeY38A10A.6, NP_504574; ScDbp1p, *Saccharomyces cerevisiae* Dbp1p, NP_015206; and AraNM_126777, *Arabidopsis thaliana* putative helicase, NP_178818.G.

DDX3 was identified initially as a putative DEAD-box helicase [15], and two almost identical genes, for DDX3X and DDX3Y (91.7% homology at the amino acid level), were found on the human X chromosome and the human Y chromosome, respectively. Mutations in human DDX3Y are a frequent cause of male infertility, suggesting an essential role for DDX3Y in embryogenesis [16]. Recently it has been reported that DDX3 is an RNA-dependent ATPase/helicase that shuttles between the nucleus and the cytoplasm, functioning in the Rev-RRE/CRM1 pathway for the export of unspliced and partially spliced HIV-1 transcripts [17]. Homologs of the gene for DDX3 proteins have been found not only in mammals but also found in other species: *Ded1p* in yeast, *Bel1* in *Drosophila*; and *mut-14*, and *vbh-1* in *Caenorhabditis elegans* (Figure 4).

**Figure 5:**
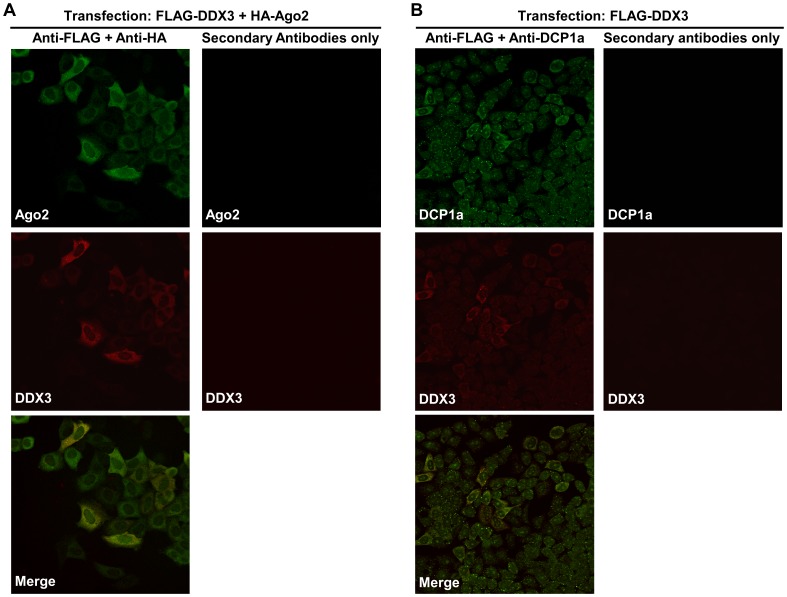
Colocalization of DDX3 with Ago2 in HeLa S3 cells. (A) Subcellular localization of Flag-tagged DDX3 (Red) and HA-tagged Ago2 (Green) in HeLa S3 cells, as determined by anti-Flag and anti-HA immunofluorescence microscopy. (B) Flag-tagged DDX3 is colocalized with the P-body marker DCP1a in P-body granules.

We examined the subcellular localization of DDX3 in HeLa S3 cells and analyzed its colocalization with RISC and P-body using expression vectors that encoded Flag-tagged DDX3 and HA-tagged Ago2, and a monoclonal antibody against P-body marker, DCP1a. As shown in Figure 5, DDX3 and Ago2 were colocalized in the cytoplasm. This observation suggests that DDX3 might act with or near RISC.

**Figure 6:**
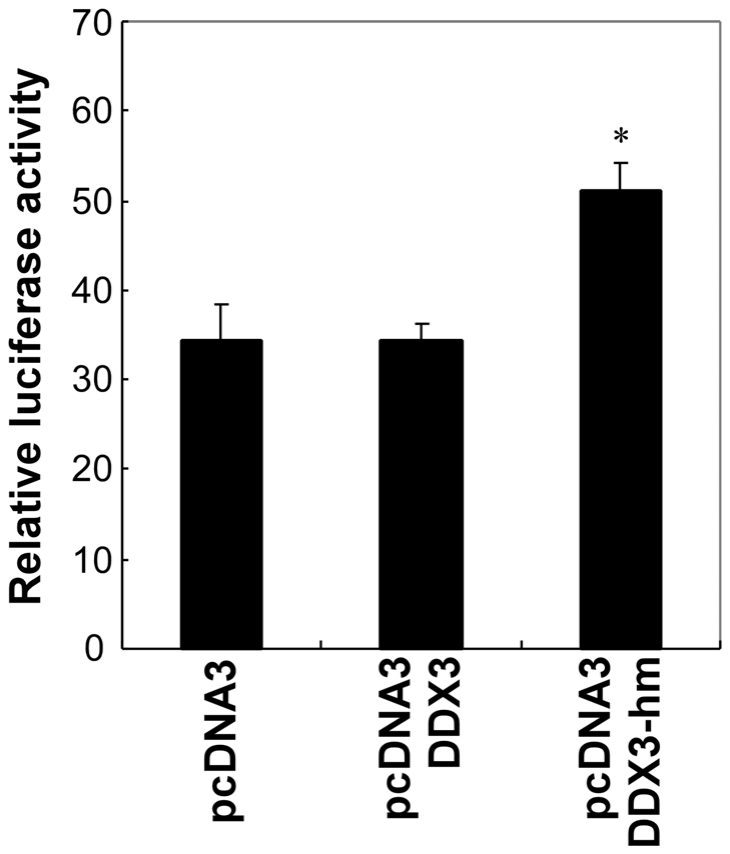
Effect of a dominant-negative mutant of DDX3 on RNAi. HeLa S3 cells were transfected with 250 ng of the DDX3-expression vector or DDX3 mutant expression vector, 10 ng of the shRNA vector directed against firefly luciferase, 25 ng of the firefly luciferase expression vector, and 2.5 ng of the *Renilla* luciferase-expression vector. Cells were harvested 24 h after transfection and assayed for luciferase activity. Data was shown as mean and S.D. (n = 3). *P<0.05.

To determine whether the activity of DDX3 is critical for RNAi, we created helicase activity-deficient DDX3 by mutating lysine at position 230 to glutamic acid [17], and then we examined the dominant-negative effect of the mutant on RNAi. In cells that expressed dominant-negative DDX3 (pcDNA3 DDX3-hm), RNAi activity was lower than in control cells and in DDX3-expressing cells (pcDNA3 DDX3) (Figure 6). These observations implied that the activity of DDX3 might play an important role in RNAi.

Genome-wide analysis of RNAi pathway in *C. elegans* by Kim et al. [18] indicated that Y38A10A.6, which is one of the homologs of DDX3 in *C. elegans* (Figure 4), has an indispensable role. Furthermore, recently, Pek et al. [19] reported that DDX3 and its homolog in *Drosophila* somatic cells, Belle, are required in chromosal localization of hCAP-H and Barr, respectively, and thus promoting chromosome segregation, in a Dicer and Ago2 dependent manner. These evidences support that DDX3 orthologs are crucial for RNAi pathway, but this has never been tested in mammals. Thus, our result is the first one to demonstrate the essential role of DDX3 in mammalian RNAi pathway.

In summary, in this study, we have identified DDX3 as an essential factor involved in mammalian RNAi pathway by screening of an shRNA-expression library that we generated previously [12]. Immunohistochemical analysis revealed that DDX3 is colocalized with Ago2, an essential component of RISC which is also known as a marker of P-body [20], indicating that DDX3 might be localized in the rough endoplasmic reticulum and P-bodies [21], [22]. Furthermore, results of experiments with a dominant negative mutant of DDX3 further confirmed that this gene affects the RNAi activity.

In conclusion, we have identified DDX3 as a crucial factor for RNAi in mammalian cells using our shRNA-expression library. Further detailed analysis, including the analysis of other positive clones obtained during our screening should help us to provide additional details of the RNAi machinery for its action and the processing mechanisms that generate non-coding RNAs.
